# The association between Healthy Beverage Index and psychological disorders among overweight and obese women: a cross-sectional study

**DOI:** 10.1186/s12905-022-01870-3

**Published:** 2022-07-15

**Authors:** Niloufar Rasaei, Rasool Ghaffarian-Ensaf, Farideh Shiraseb, Faezeh Abaj, Fatemeh Gholami, Cain C. T. Clark, Khadijeh Mirzaei

**Affiliations:** 1 Department of Community Nutrition, School of Nutritional Sciences and Dietetics, Tehran University of Medical Sciences (TUMS), P.O. Box: 14155-6117, Tehran, Iran; 2 Department of Nutrition, Science and Research Branch, Islamic Azad University, Tehran, Iran; 3 Centre for Intelligent Healthcare, Coventry University, Coventry, CV1 5FB UK; 4 Food Microbiology Research Center, Tehran University of Medical Sciences, Tehran, Iran

**Keywords:** Depression, Anxiety, Stress, Mental health, Healthy beverage index, Obesity

## Abstract

**Background and aims:**

The Healthy Beverage Index (HBI) is a valuable technique to estimate the synergistic effects of overall beverage consumption. Several studies have evaluated the associations between HBI and beneficial changes in the health status. however, there is no study on the association between patterns of beverage consumption and mental health status. Therefore, this study sought to examine the association between HBI and psychological disorders among overweight and obese women.

**Methods:**

199 overweight and obese women, between the ages of 18 and 55 y, were enrolled in this cross-sectional study in Tehran, Iran. To collect beverage dietary data, a validated semi-quantitative food-frequency questionnaire (FFQ) was used. Furthermore, the DASS-21 questionnaire was used to assess psychological profile states.

**Results:**

The association of total depression anxiety stress (DASS) score with healthy beverage index (HBI) tertiles in models was marginally significant (OR =: 0.78; 95% CI 0.30–2.02; *P*-value = 0.074; (OR = 0.77; 95% CI 0.28–2.16; *P*-value = 0.062), respectively. In terms of stress, anxiety, and depression, after adjusting for confounders, participants with higher HBI in the third tertile had lower odds of depression vs. the first tertile (OR = 0.99; 95% CI 0.35–2.81; *P*-trend = 0.040).

**Conclusion:**

We demonstrate that the total DASS score was associated with HBI tertiles. We also found that participants with higher HBI had lower odds of depression. However, additional well-designed studies are needed to confirm the veracity of these findings.

## Introduction

According to the World Health Organization, psychological health issues are expected to become an increasingly serious risk to public health by 2030 [1]. Psychological distress such as depressionn and anxiety, is among the most prominent conditions that compose mental diseases [2]. Depression is a severe mental illness that affects around 4.4% of the world's population, which is a significant leading cause of disease burden [3]; furthermore, anxiety affects about one in 13 people of the population [4]. These psychiatric problems not only have negative occupational, academic, and social consequences, but they also create a major financial burden on the medical system [5, 6].

Psychological problems are becoming more common, especially in women, who are twice as likely as males to suffer from depression [7, 8]. Obesity and psychological illnesses have a direct association; indeed, obesity can lead to psychological illnesses such as depression and anxiety. Obesity has been shown to increase the prevalence of depression by 32.8 percent and anxiety disorder by 30.5 percent [9]. According to research carried out by Noorbala and colleagues in 2004 using the General Health Questionnaire, 21 percent of the overall population in Iran suffered from mental health problems (25.9% of women and 14.6 percent of males) [10]. In 2008, this percentage was raised to 36% in Tehran, with women showing a greater percent compared to males (2 to 1) [11].Moreover, Obesity patterns in different nations are based on gender. Women are more likely than males to be diagnosed with obesity [12, 13]. Given the relevance and high frequency of mental health disorders, particularly among women, as well as their greatest influence in generating other diseases and consequences [14], observational studies to assess the risk of psychological disorders in overweight and obese women should be developed to improve mental health. Adults who have been classified with depression or anxiety are also much more likely to be inactive and obese [15]. Both mental disorders and obesity are affected by a variety of inherited and environmental variables [16–18], and diet has been proposed as a key component in the development of these prevalent diseases [19–21].

To date, substantial research has been conducted on the relationship between individual beverage consumption and overweight/obesity, particularly sugar-sweetened beverages [22–24]. Milk, coffee, tea, and other unsweetened beverages have been linked to improved health, particularly cognitive function [25, 26]. Sugar-sweetened beverage (SSB) consumption, on the other hand, has been linked to weight gain and obesity [27–29].

Few studies, however, have investigated the general quality of daily beverage consumption in the context of evaluating all consumed beverages as a pattern studies [30–33]. There is also another study which validated HBI in Iran [34]. A Healthy Beverage Index (HBI) could be used to assess overall beverage quality and to discern whether changes in beverage consumption patterns are related to better health. As a result, the HBI has been established as a comprehensive concept in nutritional epidemiology studies to evaluate the quality of overall beverage intake and its association with health-related outcomes [30]. Eight beverage categories, total beverage energy, and fluid intake were included in this index [35]. The Beverage Guidance Panel provided the majority of HBI components; however, the recommendations were transformed in to fluid requirements as a percentage of overall fluid requirements. Furthermore, the HBI classified "caloric drinks with certain nutrients" into three categories: full fat milk, 100% fruit juice, and alcohol. The usual fluid requirement of one mL per kcal eaten was used to calculate total fluid requirements [36].Because the effect of a single beverage may be too weak to be identifiable, the cumulative effects of numerous beverages incorporated in a total dietary index may give better identification [26]. Furthermore, because the HBI can be used as a counseling tool to encourage healthy beverage selection, it may have significant ramifications for public health.

To the best of our knowledge, no study has investigated the relationship between HBI patterns and the risk of psychological disorders in overweight and obese women. Several studies have suggested that adherence to the HBI might improve the abnormal plasma lipid markers and other risk factors associated with metabolic syndrome and cardiovascular disease (CVDs) [30, 34], whilst some studies have demonstrated HBI scores are associated with beneficial changes in the health status of women. Considering some of the risk factors for psychological disorders, and the possible association between the HBI diet and these risk factors, we conducted this study to determine the association between HBI dietary patterns and the risk of psychological disorders in overweight and obese women.

## Method and materials

### Study population

Participants were chosen from a cross-sectional survey conducted in Tehran, Iran, in 2019. This study enrolled a total of 199 obese and overweight women between the ages of 18 and 55. Women with a history of CVDs, hypertension, type 2 diabetes (T2D), polycystic ovary syndrome (PCOS), kidney failure, stroke, thyroid disease, liver disease, cancer, inflammatory disorders, and individuals taking any therapeutic drugs, weight loss program, or supplements during the study period were excluded. Another exclusion criterion included total energy consumption of < 500 or > 3500 kcal/day. To take part in our study, all participants signed a written informed consent form prior to study commencement.

### Data collection

Participants' age, marital status, and educational level were recorded. For measuring the height, we used a non-stretch tape measure, with participants in a standing position and unshod, height was measured and recorded at a precision of 0.5 cm [37]. With the individuals standing upright, NC was measured using non-stretchable plastic tape at the halfway point of the neck, between the mid-cervical spine and the mid anterior neck, to within 1 mm. Body mass index (BMI) was determined by dividing body weight by the square of body height and is represented in kilograms per square meter (kg/m^2^), with weight in kilograms and height in meters. A manual sphygmomanometer was used to monitor systolic and diastolic blood pressure on the left arm, while sitting, after a 5-min rest interval. The Tehran University of Medical Sciences (TUMS) Ethics Committee approved this study (IR.TUMS.MEDICINE.REC.1401.206). All methods were performed in accordance with relevant guidelines and regulations.

### Physical activity assessment

Individuals' physical activity was assessed using the short-term International Physical Activity Questionnaire (IPAQ) [38]. This questionnaire calculates the physical activity of all participants during the past 7 days. The validity and reliability of IPAQ questionnaire was assessed across 12 countries. The criterion reliability of this questionnaire had the Spearman’s ρ of around 0.8. The median ρ for the validity has been reported at around 0.30, which was similar to other validation studies. IPAQ is a validated self-reported seven-item measure of physical activity that shows physical activity rate (vigorous, moderate, walking, and inactive) over the last week, and then the values were multiplied by their metabolic equivalent (MET) quantities, and the acquired numbers were summed together to calculate a MET/min/week value.

### Body composition

Weight (kg), fat-free mass, bone mineral content, percent body fat (percent BF), skeletal muscle mass (SMM), soft lean mass (SLM), fat-free mass index (FFMI), intracellular water, and extracellular water were all measured using a tetrapolar bioelectrical impedance analysis (InBody 770 scanner, Seoul, Korea) [20]. Participants took off their shoes, jackets, and sweaters before standing barefoot on the balancing scale and holding the machine's handles [39].

### Blood parameters

After 10–12 h night fasting, blood samples were drawn at the Nutrition and Biochemistry laboratory of the School of Nutritional Sciences and Dietetics, TUMS. Standard methods were used to collect and measure biochemical variables, including blood sugar tests (FBG and HbA1c) and lipid profiles (Triglyceride (TG) (mg/dl), high-density lipoprotein (HDL) (mg/dl), total cholesterol (TC) (mg/dl), and low-density lipoprotein (LDL) (mg/dl).

### Dietary assessment

Face-to-face interviews were used to assess dietary consumption using a standardized and reliable food-frequency questionnaire (FFQ) [40]. Subjects were ask to report the frequency of each food item consumed on a daily, weekly, monthly, or yearly during the past year. This evaluation was conducted by asking participants about the occurrence of food items consumed from a prepared list of foods. Using home measures, the final portion amounts were converted to grams per day. The residual approach was then used to modify these figures for calorie intake. Dietary intakes were assessed by the Iranian Food Composition Table (FCT) and by using N4 (First Data Bank, San Bruno, CA) software to estimate energy and nutrient intakes.

### HBI scoring system

Dufey and Davy [30] formulated a method for calculating the HBI. Water, unsweetened coffee and tea, low-fat milk (1.5 percent fat, fat-free, and/or soy milk), diet drinks (including non-calorically sweetened coffee and tea and other artificially sweetened beverages), 100 percent fruit juice, alcohol (including beer, wine, and liquor), full-fat milk (1.5 percent fat), and sugar-sweetened beverages (including fruit drinks, sweetened coffee and tea, and soda) were the eight categories of beverages consumed [30]. The final HBI score ranges from 0 to 100, with a higher number indicating better compliance with beverage standards [30]. The maximum final HBI score was 90, since diet drinks (with a score ranging between 0 and 5) and alcohol (with score numbers from 0 to 5) were not consumed by participants in this study.

### Assessment of mental health

We assessed the mental health of participants using the 21-question version of the Depression Anxiety Stress Scales (DASS-21), which has been shown to be a valid tool for the evaluation of stress, depression, and anxiety. Each of the three DASS-21 scales contains 7 items, divided into subscales with similar content [41]. DASS-21 scores were multiplied by 2 to calculate the final score, as is reported based on guidelines. Scores ≥ 10, ≥ 8, and ≥ 15 were considered as cutoff points for having depression, anxiety, and stress, respectively [42].

### Statistical analysis

SPSS v.26 software (SPSS Inc., IL, USA) was used for statistical analysis, and statistical significance was accepted at *P* < 0.05, while *P* = 0.05–0.07 was considered marginally significant in the present study. The Kolmogorov–Smirnov test was used to determine the normality of data distribution; quantitative data were reported as means and standard deviation (SD), and categorical data were reported as numbers with percentage. According to the HBI, the participants were categorized in to tertiles based on their scores, to: tertile 1 (< 63), tertile 2 (63–67), and tertile 3 (> 67), respectively. To compare quantitative and categorical variables across HBI tertiles, one-way analysis of variance (ANOVA) and chi-square (*χ*^2^) tests were performed, respectively. After controlling for confounders (age, body mass index, energy intake, physical activity), and considering BMI as a collinear variable for anthropometrics and body composition variables, dietary intakes were compared across the tertiles of HBI using analysis of covariance (ANCOVA). Binary logistic regression was used to determine whether different HBI were associated with the risk of depression, anxiety, and stress. In adjusted model 1, age, BMI, energy intake, and physical activity were controlled. In adjusted model 2, age, BMI, energy intake, physical activity, education level, job, and marital status were controlled. An odds ratio (OR) with 95% Confidence Interval (CI) was calculated.

## Results

### Study population characteristics

One hundred and ninety-nine participants completed this study, where the overall prevalence of HBI tertiles was 72 (36.2%) for tertile 1, 76 (38.2%) for tertile 2, and 51 (25.6%) for tertile 3. The mean (SD) age and BMI of participants were 36.09 (8.52) years and 30.77 (4.22) kg/m^2^, respectively. The economic status, marriage, and employment were such that 77 (38.7%) respondents had a moderate economic status, 153 (76.9%) respondents were married, and 113 (56.8%) were employed. The majority of respondents were educated to diploma (75 (37.7%)) and bachelor or higher (98 (49.2%)) level. The mean (SD) of the DASS score, stress score, anxiety score, and depression score were 37.46 (24.56), 16.11 (10.13), 10.59 (8.24), and 10.75 (9.70), respectively.

### Baseline characteristics of study particiants categorized according to the tertiles of HBI in obese and overweight women

The baseline characteristics of study participants, categorized according to the HBI tertiles, are presented in Table 1. As shown in Table 1, *P*-values for all variables were reported in the crude model, and after adjustment with potential confounders, including age, BMI, energy intake, and PA. In the crude model, there was a significant mean difference in terms of physical activity among the tertiles of HBI (*P* = 0.044), neck circumference (NC) (*P* = 0.021), total cholesterol (TC) (0.011), high-density lipoprotein cholesterol (HDL-c) (*P* = 0.019), and low-density lipoprotein cholesterol (LDL-c) (*P* = 0.001). After adjustment with confounders (age, BMI, energy intake, and physical activity), the mean difference of fat-free mass (FFM) (*P* = 0.011), bone mineral content (BMC) (*P* = 0.022), skeletal muscle mass (SMM) (*P* = 0.011), soft lean mass (SLM) (*P* = 0.011), fat-free mass index (FFMI) (*P* = 0.036), intracellular water (IW) (*P* = 0.037), and extracellular water (EW) (*P* = 0.024) became significant. In terms of physical activity, NC, TC, HDL, and LDL, after adjustment with confounders, the mean difference of HDL and LDL remained significant (*P* < 0.05). Following Bonferroni post-hoc testing, the significant mean difference in FFM, SLM, BMC, SMM, FFMI, IW, EW, and HDL were between T1 and T2, such that the mean difference of T1 was higher than T2 in all variables except HDL, where T2 was higher than T1. In terms of LDL, the mean difference was between T1 and T3, such that the mean difference in T3 was lower than in T1. In categorical variables, a significant mean difference among the participants was seen in terms of marital status (*P* = 0.009), after controlling for confounders. There was no significant difference in terms of other variables in Table 1.

**Table 1:** Baseline characteristics of study particiants categorized according to the tertiles of healthy beverage index in obese and overweight women (n = 199)

Variables	Tertiles of HBI				
	T1 (< 63) N = 72	T2 (63–67) N = 76	T3 (> 67) N = 51	*P*-value	*P*-value*
*Demographic characteristics*					
Age (y)	35.84 (8.55)	36.55 (8.92)	35.46 (8.07)	0.771	0644
IPAQ (MET min-week)	1045.83 (1990.33)	1129.33 (1207.18)	2147.35 (3861.09)	**0.044**	0.048
*Anthropometric and body composition measurements*					
Weight (kg)	80.61 (11.27)	78.73 (13.10)	81.48 (10.08)	0.403	0.102
Height (cm)	161.83 (5.65)	160.32 (5.53)	161.88 (6.17)	0.198	0.149
WC (cm)	93.78 (16.63)	94.16 (10.98)	99.28 (21.61)	0.184	0.148
HC (cm)	114.33 (9.87)	113.25 (10.18)	115.17 (8.63)	0.590	0.697
NC (cm)	36.72 (2.17)	36.55 (2.08)	38.64 (3.20)	**0.021**	0.066
BFM (%)	33.31 (8.01)	33.10 (9.02)	34.13 (7.10)	0.781	0.639
FFM (%)	47.31 (5.59)^a^	45.85 (5.74)	47.18 (5.10)	0.229	**0.011**
BMC (g)	2.73 (0.35)^a^	2.61 (0.35)	2.69 (0.32)	0.116	**0.022**
SMM (%)	25.96 (3.31)^a^	25.14 (3.42)	25.93 (3.04)	0.253	**0.011**
SLM (%)	44.58 (5.26)^a^	43.24 (5.43)	43.87 (5.20)	0.315	**0.011**
BMI (kg/m^2^)	30.77 (3.98)	30.72 (4.71)	31.07 (3.89)	0.896	0.835
BF (%)	40.93 (5.50)	41.28 (5.16)	41.06 (6.12)	0.926	0.944
WHR	0.93 (0.05)	0.93 (0.05)	0.94 (0.04)	0.249	0.821
FFMI	18.03 (1.54)^a^	17.82 (1.60)	20.64 (18.60)	0.220	**0.036**
FMI	12.79 (3.13)	12.91 (3.50)	13.27 (3.19)	0.728	0.929
IW (L)	21.44 (2.54)^a^	20.84 (2.63)	21.43 (2.31)	0.276	**0.037**
EW (L)	13.30 (1.59)^a^	12.91 (1.67)	13.24 (1.46)	0.297	**0.024**
*Blood pressure*					
SBP (mmHg)	113.70 (11.23)	111.41 (14.20)	111.53 (13.53)	0.522	0.205
DBP (mmHg)	79.94 (8.27)	77.35 (11.35)	76.75 (9.65)	0.163	0.269
*Biochemical variables*					
FBS (mg/dl)	86.61 (9.96)	86.07 (8.78)	88.42 (10.07)	0.405	0.670
TC (mg/dl)	179.23 (31.21)	181.82 (38.41)	199.06 (40.96)	**0.011**	0.125
TG (mg/dl)	131.90 (69.16)	112 (55.55)	118.63 (60.76)	0.158	0.089
HDL_C (mg/dl)	44.25 (8.59)^a^	48.97 (11.35)	44.49 (13.16)	**0.019**	**0.033**
LDL_C (mg/dl)	98.96 (21.22)	95.53 (25.98)	81.96 (23.43)^b^	**0.001**	**0.021**
AST (IU/L)	18.07 (7.45)	17.76 (8.10)	17.98 (6.70)	0.968	0.693
ALT (IU/L)	20.31 (13.36)	20 (15.48)	18.38 (10.09)	0.732	0.778
Hs-CRP (mg/l)	5 (5.12)	4.36 (4.77)	3.81 (3.97)	0.420	0.920
Insulin (µU/ml)	1.24 (0.24)	1.22 (0.26)	1.21 (0.20)	0.812	0.579
HOMA index	3.22 (1.33)	3.43 (1.44)	3.64 (1.67)	0.265	0.535
ISQIUKI (mg/l)	0.50 (0.02)	0.50 (0.02)	0.50 (0.03)	0.714	0.865
*Economic category*			0.310	0.081	
Poor (< 5,000,000 Rials)	26 (49.1)	16 (30.2)	11 (20.8)		
Moderate					
(5,000,000–15,000,000 Rials)	26 (34.7)	33 (44)	16 (21.3)		
Good (> 15,000,000 Rials)	17 (32.7)	20 (38.5)	15 (28.8)		
*Education category*			0.953	0.377	
Illiterate	1 (33.3)	1 (33.3)	1 (33.3)		
Under diploma	8 (38.1)	8 (38.1)	8 (38.1)		
Diploma	28 (37.8)	25 (33.8)	21 (28.4)		
Bachelor and higher	34 (35.8)	40 (42.1)	21 (22.1)		
*Marital status*				0.077	**0.009**
Single	10 (23.3)	22 (51.2)	11 (25.6)		
Married	61 (40.7)	52 (34.7)	37 (24.7)		
*Supplement intake*				0.520	0.240
Yes	42 (50.6)	33 (39.8)	8 (9.6)		
No	25 (41)	29 (47.5)	7 (11.5)		
*Job category*				0.598	0.444
Employed	44 (39.3)	40 (35.7)	28 (25)		
Unemployed	26 (33.8)	33 (42.9)	18 (23.4)		

Values are represented as means (SD)

Categorical variables: N (%)

ANCOVA (*P* value*) was performed to adjusted potential confounding factors (age, BMI, energy intake, Physical activity)

BMI consider as collinear variable for anthropometrics and body composition variables

*P*-values < 0.05 were considered as significant

AC, Arm Circumference; ALT, Alanine Aminotransferase; AST, Aspartate Aminotransferase; BFM, Body Fat Mass; BFP, Body Fat Percent; BMC, Bone Mineral Content; BMI, Body Mass Index; DBP, Diastolic Blood Pressure; EW, Extracellular Water; FFMI, Fat-Free Mass Index; FFM, Fat-Free Mass; FMI, Fat Mass Index; HC, Hip Circumference; HDL_C, High-Density Lipoprotein Cholesterol; HOMA Index, Homeostatic Model Assessment Index; IW, Intracellular Water; LDL_C, Low-Density Lipoprotein Cholesterol; NC, Neck Circumference; SBP, Systolic Blood Pressure; SLM, Soft Lean Mass; SMM, Skeletal Muscle Mass; TC, Total Cholesterol; TG, Triglyceride; VFA, Visceral Fat Area; VFL, Visceral Fat Level; WC, Waist Circumference; WHR, Waist to Hip Ratio; hs CRP, High-Sensitivity C-Reactive Protein

^a^The significant difference was seen between T1 and T2

^b^The significant difference was seen between T1 and T3

### Dietary food intakes across HBI tertiles in obese and overweight women

Dietary intake of all participants according to HBI tertiles is presented in Table 2. Mean difference of vitamin E (*P* = 0.008), whole grains (*P* = 0.003), and fruits (*P* = 0.032) was statistically significant in the crude model. After controlling for energy intake, the mean difference of carbohydrate (*P* = 0.027), potassium (*P* = 0.027), and biotin (0.011) became significant (*P* < 0.05). In terms of vitamin E, whole grains, and fruits, the mean difference remained significant (*P* < 0.05).

**Table 2:** Dietary food intakes across healthy bevarage index tertiles in obese and overweight women (n = 199)

Variables	Tertiles of HBI				
	T1 (< 63) N = 72	T2 (63–67) N = 76	T3 (> 67) N = 51	*P*-value	*P*-value^a^
		Mean (SD)			
*Dietary intakes*					
Energy (kcal/d)	2575.34 (642.93)	2683.03 (802.29)	2683.03 (802.29)	0.671	–
Protein (g/d)	87.44 (25.15)	93.17 (30.69)	88.21 (29.56)	0.440	0.680
Carbohydrate (g/d)	362.34 (113.91)	374.65 (126.59)	384.89 (132.19)	0.612	**0.027**
Total fat (g/d)	94.10 (30.71)	98.79 (33.68)	88.08 (33.10)	0.211	0.085
Cholesterol (mg/d)	239.37 (98.74)	269.93 (115.50)	261.45 (97.41)	0.208	0.361
SFA (mg/d)	27.08 (9.86)	30.22 (12.46)	26.21 (11.11)	0.107	0.113
EPA (mg/d)	0.04 (0.04)	0.03 (0.04)	0.03 (0.04)	0.518	0.356
DHA (mg/d)	0.13 (0.13)	0.11 (0.12)	0.11 (0.12)	0.577	0.387
TFA (mg/d)	0.001 (0.002)	0.001 (0.002)	0.001 (0.004)	0.660	0.662
Sodium (mg/d)	4272.31 (1288.95)	4406.65 (1435.55)	3996.72 (1384.17)	0.275	0.207
Potassium (mg/d)	4197.62 (1557.12)	4420.46 (1490.27)	4741.48 (1713.63)	0.183	**0.027**
Vitamin A (RAE)	737.27 (347.20)	852.35 (429.49)	817.10 (411.53)	0.212	0.347
Β-carotene (mg/d)	4832.23 (2643.14)	5848.37 (3727.51)	5382.52 (3046.74)	0.165	0.245
Vitamin D (µg/d)	2.08 (1.65)	1.85 (1.37)	2.13 (1.69)	0.549	0.406
Vitamin E (mg/d)	19.63 (11.19)	15.25 (6.38)	15.51 (9.11)	**0.008**	**0.002**
Thiamin (µg/d)	2.06 (0.62)	2.09 (0.65)	2.04 (0.74)	0.920	0.615
Riboflavin (mg/d)	2.19 (0.87)	2.25 (0.73)	2.30 (0.97)	0.765	0.576
Niacin (mg/d)	24.43 (7.10)	26.29 (9.82)	25.56 (10.73)	0.473	0.695
Vitamin B6 (µg/d)	2.07 (0.63)	2.23 (0.76)	2.29 (0.77)	0.220	**0.065**
folate (µg/d)	597.78 (175.03)	612.84 (172.03)	620.89 (190.04)	0.768	0.497
Vitamin B12 (µg/d)	4.22 (1.92)	4.70 (2.48)	4.72 (3.52)	0.461	0.564
Biotin	37.17 (15.08)	38.21 (13.30)	45.04 (26.52)	0.051	**0.011**
Pantothenic acid	6.47 (2.15)	6.34 (1.90)	6.99 (3.81)	0.381	**0.068**
Vitamin K (µg/d)	190.24 (108.55)	246.13 (212)	214.33 (124.93)	0.111	0.154
Phosphorus (mg/d)	1630.50 (488.95)	1692.39 (536.33)	1636.74 (572.29)	0.752	0.981
Vitamin C µmol/L)	184.68 (161.52)	185.51 (102.52)	229.87 (132.41)	0.139	**0.052**
Calcium (mg/d)	1155.53 (416.47)	1193.74 (411.57)	1156.70 (449.51)	0.837	0.981
Iron (mg/d)	18.37 (5.74)	19.26 (5.91)	18.77 (7.03)	0.689	0.953
Magnesium (mg/d)	455.68 (143.05)	475.01 (148.35)	476.58 (165.83)	0.677	0.566
Zinc (mg/d)	13 (4.23)	13.42 (4.22)	13.14 (4.83)	0.839	0.963
Copper (mg/d)	1.93 (0.60)	2.05 (0.62)	2.13 (1.07)	0.329	0.105
Manganese (mg/d)	7.12 (2.37)	7.38 (2.80)	7.23 (3.83)	0.875	0.999
Selenium (mg/d)	121.30 (36.19)	120.46 (43.10)	117.03 (50.83)	0.859	0.430
Chromium (mg/d)	0.12 (0.07)	0.11 (0.09)	0.10 (0.09)	0.419	0.337
Caffeine (mg/d)	141.48 (104.62)	177.19 (129.08)	180.36 (288.04)	0.380	0.403
*Food groups*					
Whole grains (g/d)	6.58 (7.94)	7.35 (11.18)	13.44 (15.09)	**0.003**	**0.003**
Fruits (g/d)	494.29 (376.17)	499.23 (304.22)	655.79 (413.98)	**0.032**	**0.004**
Vegetables (g/d)	429.44 (261.88)	464.14 (252.50)	495.74 (325.18)	0.431	0.471
Nuts (g/d)	13.66 (17.01)	18.23 (17.40)	16.61 (19.86)	0.307	0.323
Legumes (g/d)	55.74 (45.17)	56.49 (41.62)	53.97 (43.68)	0.952	0.956
Tea and coffee (ml/d)	684 (517.17)	868.44 (628.04)	930.27 (1453.97)	0.268	0.311
Refined grains (g/d)	444.62 (239.25)	411.84 (195.06)	428.32 (280.15)	0.707	0.279
SSB (ml/d)	14.47 (22.73)	28.54 (81.47)	13.20 (29.50)	0.196	0.232
Dairy (ml/d)	396.12 (248.83)	376.14 (214.52)	425.73 (331.72)	0.597	0.364
Eggs (g/d)	19.87 (12.51)	21.60 (13.42)	24.96 (15.34)	0.136	0.156
Fish and seafood (g/d)	12.82 (12.72)	11.41 (10.64)	12.36 (13.61)	0.782	0.554
Meat (g/d)	58.49 (39.08)	76.85 (62.76)	64.55 (41.94)	0.086	0.150
Red meat (g/d)	19.19 (14.89)	26.60 (23.01)	22.34 (21.22)	0.085	0.128
Low fat dairy (ml/d)	312.58 (246.75)	249.37 (173.01)	324.66 (303.32)	0.158	0.087
High fat dairy (ml/d)	86.32 (111.77)	128.14 (163.88)	101.98 (129.29)	0.192	0.299
Poultry (g/d)	31.66 (29.29)	42.77 (49.13)	33.56 (26.08)	0.176	0.264

Values are represented as means (SD)

ANCOVA (*P* value*) was performed to adjusted potential confounding factors (energy intake)

*P*-values < 0.05 were considered as significant

DHA, Docosahexaenoic Acid; EPA, Eicosapentaenoic Acid; SFA, Saturated Fatty Acid; SSB, Sugar Sweetened Beverages; TFA, Trans Fatty Acid

There was no significant difference in other variables in Table 2.

### Association of psychological disorders and healthy beverage index in obese and overweight women

The associations of stress, anxiety, depression, and total DASS score with HBI are shown in Table 3. In the crude model, there was no significant relationship between depression, anxiety, stress, and total DASS score with the tertiles of HBI (*P* > 0.05). After adjusting for confounders in model 1 (adjusting for age, energy intake, BMI, and physical activity) and model 2 (adjusting for age, energy intake, BMI, physical activity, marital status, economic status, job, and education), the mean difference of variables remained insignificant (*P* > 0.05).

**Table 3:** Association of psychological disorders and healthy beverage index in obese and overweight women (n = 199)

Mental health	Tertiles of HBI			
Depression	T1 (< 63) N = 72	T2 (63–67) N = 76	T3 (> 67) N = 51	*P*-value
Crude	5.62 (5.12)	5.14 (4.45)	5.37 (5.11)	0.836
Model 1	5.58 (0.65)	5.15 (0.65)	4.99 (0.80)	0.839
Model 2	5.60 (0.66)	5.52 (0.69)	5.04 (0.87)	0.882
*Anxiety*				
Crude	4.55 (3.68)	5.51 (4.49)	6.02 (4.03)	0.128
Model 1	4.72 (0.53)	5.65 (0.54)	5.46 (0.66)	0.458
Model 2	4.73 (0.54)	5.93 (0.58)	5.36 (0.72)	0.323
*Stress*				
Crude	7.83 (4.91)	7.84 (5.23)	8.69 (5.08)	0.590
Model 1	7.39 (0.69)	8.26 (0.70)	8.60 (0.86)	0.540
Model 2	7.42 (0.68)	8.64 (0.72)	8.49 (0.91)	0.442
*Total DASS score*				
Crude	18.01 (12.13)	18.5 (12.25)	20.08 (12.67)	0.644
Model 1	17.70 (1.65)	19.05 (1.66)	19.06 (2.05)	0.822
Model 2	17.75 (1.65)	20.09 (1.75)	18.89 (2.20)	0.624

Total DASS score: Total Depression Anxiety Stress Scales score

*P*-values < 0.05 were considered as significant

*P* value with unadjusted (crude)

Adjusted model 1: Adjusted for age, energy intake, BMI, physical activity

Adjusted model 2: Adjusted for age, energy intake, BMI, physical activity, marital status, economic status, job, education

Data in crude model are presented as mean (SD)

Date in model 1 and model 2 are presented as mean (SE)

### Association of DASS score and it’s components with HBI tertiles in obese and overweight women

The crude and adjusted OR and 95% CI of the DASS score and its components across tertiles of HBI were shown in Table 4. In the crude model, there was no significant association between DASS score and its components with HBI tertiles (*P* > 0.05). After adjustment with confounders in model 1 (adjusting for age, energy intake, BMI, and physical activity) and model 2 (adjusting for age, energy intake, BMI, physical activity, marital status, economic status, job, and education), the association of total DASS score with HBI tertiles was marginally significant (OR 0.78; 95% CI 0.30–2.02; *P*_value = 0.074), (OR 0.77; 95% CI 0.28–2.16; *P*_value = 0.062), respectively. In terms of stress, anxiety, and depression, after adjusting with confounders in model 2, participants with higher HBI (third vs. first tertile) had lower odds of depression (OR 0.99; 95% CI 0.35–2.81; *P*_trend = 0.040).

**Table 4:** Association of DASS score and it’s components with healthy bevarage index tertiles in obese and overweight women (n = 199)

Variables	T1 (< 63) N = 72	T2 (63–67) N = 76	T3 (> 67) N = 51	*P*-trend
Stress				
Crude	Ref	0.98 (0.48, 2.01)	0.99 (0.48, 2.01)	0.966
*P*-value		0.964	0.970	
Model 1	Ref	0.97 (0.36, 2.62)	1.01 (0.40, 2.52)	0.945
*P*-value		0.954	0.980	
Model 2	Ref	0.86 (0.29, 2.57)	1.11 (0.39, 3.12)	0.724
*P*-value		0.794	0.844	
Anxiety				
Crude	Ref	0.69 (0.33, 1.43)	0.93 (0.46, 1.91)	0.297
*P*-value		0.317	0.852	
Model 1	Ref	0.92 (0.35, 2.36)	1.26 (0.52, 3.04)	0.772
*P*-value		0.856	0.610	
Model 2	Ref	0.99 (0.35, 2.79)	1.51 (0.55,4.12)	0.843
*P*-value		0.994	0.425	
Depression				
Crude	Ref	0.95 (0.46, 1.95)	0.86 (0.42, 1.76)	0.928
*P*-value		0.893	0.686	
Model 1	Ref	1.31 (0.49, 3.52)	1.01 (0.40, 2.55)	0.059
*P*-value		0.591	0.987	
Model 2	Ref	1.14 (0.39, 3.29)	0.99 (0.35, 2.81)	0.040
*P*-value		0.811	0.993	
Total DASS score				
Model 1	Ref	0.78 (0.30, 2.02)	0.91 (0.37, 2.21)	0.603
*P*-value		0.074	0.831	
Model 2	Ref	0.77 (0.28, 2.16)	0.99 (0.36, 2.75)	0.572
*P*-value		0.062	0.996	

Binary logistic regression was used

Tertile 1 consider as a reference group

Data are presented as odds ratio (OR) and (95% confidence interval)

*P*-values < 0.05 were considered as significant

*P*-trend < 0.05 were considered as significant

*P* value with unadjusted (crude)

Adjusted model 1: Adjusted for age, energy intake, BMI, physical activity

Adjusted model 2: Adjusted for age, energy intake, BMI, physical activity, marital status, economic status, job, education

DASS: Depression Anxiety Stress Scales, HBI: Healthy Beverage Index

## Discussion

We investigated the association between HBI and psychological disorders among overweight and obese women. To the best of our knowledge, no studies have been conducted in this field thus far, and this study represents the first contribution to the literature in this regard. In the present study, total DASS score was associated with the second tertile of HBI; meaning that with increasing HBI score, the total DASS score decreased. We also found that participants with higher HBI had lower odds of depression. Based on our results, beverages might impact on micro and macronutrients. As explained in other studies and consistent with our results, beverages like milk can impact on macro and micronutrients (like potassium) [43, 44].

In one study, daily consumption of sugar sweetened-beverages (SSBs) contributed to manifestation of psychological disorders; however, no association was observed between consuming 100% fruit juice and psychological disorders [45]. In this study, only SSBs and 100% fruit juice were examined, and the authors did not categorize their results based on DASS scores. Consistent with our results, some studies have reported that the amount of sugar from beverages is associated with a higher incidence of depression and other psychological disorders [46]. It should be noted that 66.9% of participants were men while in our study the participants were women. It was demonstrated that overconsumption of SSBs caused dysregulation of the stress response [47]. In another study, it was demonstrated that the risk of depression in subjects consuming 3 cups of tea daily is 37% lower than in those who do not consume tea [48]. In this study, coffee consumption acted as tea and reduced depression. Numerous studies, conducted in China [49], Singapore [50], and USA [51], have shown a significant association between caffeine and caffeinated drinks consumption and depression. Indeed, a study conducted in the USA on women with a mean age of 63 demonstrated that women who drink more than four cups of caffeinated coffee had 20% lower risk of depression than women who consumed less [51]. These studies were consistent with our study. In two studies conducted in Finland [52, 53], the relationship between tea consumption and depression was weak or not significant. It seems that the difference between these weak results could be due to the difference in the gender and age of participants, as well as the different scales that were used for the evaluation of depression. A study conducted on children found that depressed children consume more caffeinated drinks than children with non-depressed symptoms [54] this study were inconsistent with our results. A case–control study found high consumption of soft drinks and industrial fruit juices was associated with an increased risk of depression. It should be noted that this study evaluated healthy and unhealthy dietary patterns and did not focus just on the beverages [55]. Another beverage that could be associated with depression is alcohol. According to the extant literature, people who drink alcohol are more vulnerable to depression, whilst people with depression are more likely to have alcohol misuse to relieve their distress [56–58].

The SSBs could affect mental health by their sugary components; indeed, sugar can induce chronic systematic inflammation by activating the innate immune system, thus affecting psychological disorders [59]. Animal studies have shown that sugar could increase depression incidence by activating the hypothalamic–pituitary–adrenal (HPA) axis and inducing elevation in glucocorticoids [60]. The mechanism behind the effect of coffee on the mental health is the potential stimulation of the central nervous system, enhancing dopaminergic neurotransmission [61]. One mechanism by which beverages could affect psychological disorders via carbohydrates. By modulating plasma concentrations of tryptophan and of large neutral amino acids (LNAA), carbohydrates could affect mental performance [62]. The ability of carbohydrates to increase the uptake of circulating tryptophan into the brain depends on its ability to promote the secretion of insulin [63]; thus, by consuming more carbohydrates, a greater secretion of insulin will ensue, plasma levels of LNAA will decrease, and the supply of tryptophan to the brain will increase [63]. Moreover, by consuming SSBs, and the side effects that follow, including obesity and type 2 diabetes (T2D), researchers have found a bi-directional relationship between obesity or T2D and depression [64–66].

Although representing the first study in this field, our study had some limitations that warrant consideration. First, due to the cross-sectional design, causality could not be inferred. For example, psychological disorders like depression may lead to a higher HBI score — an association that cannot be identified with our design. Second, the sample size used to conduct this study was small and should be up-scaled in further work. Third, we could not adjust for potential cofounders such as other nutrients or other demographic data, thus precluding a completely robust set of models. Nevertheless, despite the noted limitations, our study had numerous strengths, including; we assessed the association between HBI and psychological disorders among overweight and obese women for the first time, thereby allowing novel insight into this relationship; furthermore, we used a validated and reliable FFQ to evaluate the dietary intakes of the participants.

## Conclusion

We found participants with higher HBI scores had lower odds of depression. Since previous studies were consistent with our results, this is important regarding the development of preventative approaches to reduce psychological disorders. Our results showed that the total DASS score was probably associated with HBI tertiles. Based on our results and future studies in this field, it has been possible to reduce psychological disorders and especially depression, by using healthy beverages. Future studies with a bigger sample size, considering men and women, are needed to confirm the veracity of these findings.

## Data Availability

The datasets generated and /or analyzed during the current study are not publicly available due to preserving participant anonymity but are available from the Khadijeh Mirzaei on reasonable request.

## Abbreviations

BIA: Bioelectrical impedance analyzer
BMI: Body mass index
BMC: Bone mineral content
CVDs: Cardiovascular disease
DASS: Depression anxiety stress
EW: Extracellular water
FFQ: Food frequency questionnaire
FFM: Fat free mass
FFMI: Fat-free mass index
GOD-PAP: Glucose oxidase-phenol 4-aminoantipyrine peroxidase
GPOPAP: Glycerol-3-phosphate oxidase-phenol 4-aminoantipyrine peroxidase
HBI: Healthy Beverage Index
HDL: High-density lipoprotein
IW: Cholesterol intracellular water
HPA: Hypothalamic-pituitary-adrenal
LDL: Low-density-lipoprotein
LNAA: Large neutral amino acids
MET: Metabolic equivalent
NC: Neck circumference
PCOS: Polycystic ovary syndrome
IPAQ: Physical Activity Questionnaire
PA: Physical activity
SSB: Sugar-sweetened beverage
SMM: Skeletal muscle mass
SD: Standard deviation
TC: Total cholesterol
TG: Triglyceride
WC: Waist circumferences
W.H.R: Waist to hip ratio
